# Oxidative stress index as a novel biochemical marker in tuberculosis; with therapeutic benefit of antioxidant supplementation

**DOI:** 10.1186/1471-2334-12-S1-P66

**Published:** 2012-05-04

**Authors:** Suresh Babu Kondaveeti, Vamseedhar Annam, DR Suresh

**Affiliations:** 1 Department of Biochemistry, Melmaruvathur Adhiparasakthi Institute of Medical Sciences & Research, Melmaruvathur, Tamil Nadu, India; 2 Department of Pathology, Melmaruvathur Adhiparasakthi Institute of Medical Sciences & Research, Melmaruvathur, Tamil Nadu, India; 3 Department of Biochemistry, ESI-PGIMSR, Bangalore, Karnataka, India

## Background

Severe oxidative stress has been reported in tuberculosis [TB] patients. There are no previous reports regarding oxidative stress index and supplementation of antioxidants in TB. The objective of this study was to assess the antioxidant status, lipid peroxidation and oxidative stress index in TB patients. Also to assess the antioxidant supplementation with antituberculosis therapy in TB outcome.

## Methods

The present cross-sectional study comprised of normal human volunteers (n=50), untreated TB patients (n=50), TB patients treated with anti-tuberculosis therapy (n=50) and TB patients treated with combination of antioxidant supplementation with ATT (n=50). Evaluation of lipid peroxidation was performed by estimating serum malondialdehyde, while the marker for total antioxidant capacity was performed by ferric reducing antioxidant power assay. The oxidative stress index was measured. The stastical analysis performed was One Way ANOVA.

## Results

The oxidative stress index was increased significantly in untreated TB (P<0.05); these levels decreased significantly with clinical improvement in patients treated with combination of antioxidants and ATT when compared with patients treated with ATT only. The total antioxidant capacity was decreased significantly in untreated TB (P<0.05); these levels increased significantly in patients treated with combination of antioxidants and ATT when compared with patients treated with ATT only.

## Conclusion

The oxidative stress index significantly increased in untreated TB patients and decreased in TB patients on ATT with antioxidant supplementation. Hence, oxidative stress index can be considered as a novel marker in TB patients. Also, the antioxidant supplementation in adjunct with ATT showed better improvement in outcome of TB.

